# Research progress on environmental stability of SARS-CoV-2 and influenza viruses

**DOI:** 10.3389/fmicb.2024.1463056

**Published:** 2024-10-31

**Authors:** Ling Zhang, Zhongbiao Fang, Jiaxuan Li, Zhiwei Huang, Xiaotian Tie, Hongyu Li, Jianhua Li, Yanjun Zhang, Yuanyuan Zhang, Keda Chen

**Affiliations:** ^1^Key Laboratory of Artificial Organs and Computational Medicine in Zhejiang Province, Shulan International Medical College, Zhejiang Shuren University, Hangzhou, China; ^2^School of Laboratory Medicine and Life Sciences, Wenzhou Medical University, Wenzhou, China; ^3^Zhejiang Chinese Medical University, Hangzhou, China; ^4^Department of Microbiology, Zhejiang Provincial Center for Disease Control and Prevention, Hangzhou, China; ^5^Children’s Hospital, Zhejiang University School of Medicine, National Clinical Research Center for Child Health, Hangzhou, China

**Keywords:** SARS-CoV-2, influenza, environmental stability, disinfectants, environmental contamination

## Abstract

We reviewed research on SARS-CoV-2 and influenza virus detection on surfaces, their persistence under various conditions, and response to disinfectants. Viral contamination in community and healthcare settings was analyzed, emphasizing survival on surfaces influenced by temperature, pH, and material. Findings showed higher concentrations enhance survivability at room temperature, whereas stability increases at 4°C. Both viruses decline in low pH and high heat, with influenza affected by salinity. On various material surfaces, SARS-CoV-2 and influenza viruses demonstrate considerable variations in survival durations, and SARS-CoV-2 is more stable than influenza virus. On the skin, both virus types can persist for ≥2 h. Next, we delineated the virucidal efficacy of disinfectants against SARS-CoV-2 and influenza viruses. In daily life, exposure to ethanol (70%), isopropanol (70%), bleach (10%), or hydrogen peroxide (1–3%) for 15–30 min can effectively inactive various SARS-CoV-2 variants. Povidone-iodine (1 mg/mL, 1 min) or cetylpyridinium chloride (0.1 mg/mL, 2 min) may be used to inactive different SARS-CoV-2 variants in the mouth. Chlorine disinfectants (500 mg/L) or ultraviolet light (222 nm) can effectively inhibit different SARS-CoV-2 variants in public spaces. In conclusion, our study provides a scientific basis and practical guidance for reduction of viral persistence (retention of infectivity) on surfaces and environmental cleanliness.

## Graphical abstract

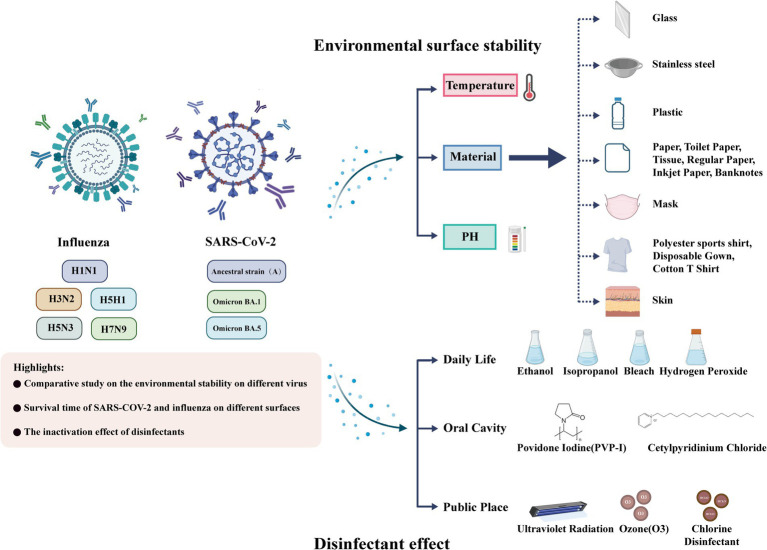

## 1 Introduction

On December 31, 2019, the World Health Organization (WHO) issued an emergency alert in response to a cluster of unexplained pneumonia cases in Wuhan, China, signaling the rapid spread of pneumonia caused by severe acute respiratory syndrome (SARS) coronavirus 2 (SARS-CoV-2), a novel coronavirus, worldwide (Li C. et al., 2022; Riou and Althaus, 2020). As the epidemic evolved into a pandemic, on February 11, 2020, the WHO officially designated this new illness as coronavirus disease 2019 (COVID-19). Throughout the main phase of the COVID-19 pandemic, SARS-CoV-2 disseminated globally, in the form of multiple variants; for instance, in December 2020, Alpha (B.1.1.7), Beta (B.1.351), and Gamma (P.1) variants were identified in the United Kingdom, South Africa, and Brazil, respectively (Sanches et al., 2021). Moreover, the Delta variant, which was concurrently discovered in India, rapidly led to the second wave of the COVID-19 pandemic in India and precipitated numerous clusters of cases in various countries and regions including the United States, exacerbating the crisis (WHO, 2024a). In November 2021, the Omicron variant was initially detected at a surveillance laboratory in South Africa, which then rapidly disseminated to numerous countries worldwide (WHO, 2024a). As of January 28, 2024, the WHO data indicate that the cumulative total cases of SARS-CoV-2 infections had increased to approximately 774 million, with the total number of deaths reaching approximately 7.0264 million (WHO, 2024a). Moreover, in January 2024, the WHO reported approximately 500,000 new COVID-19 cases and 10,000 COVID-19 deaths. These statistics highlight the severity of the COVID-19 pandemic, emphasizing the need for global prevention and control strategies, including strengthened vaccination efforts (WHO, 2024b).

Similar to the COVID-19 pandemic, that of influenza remains a major concern (Al-Qahtani, 2020; Ma et al., 2020; Zhang et al., 2020). Both SARS-CoV-2 and influenza viruses demonstrate similar transmission modes: they mainly spread through aerosol transmission, via respiratory droplets from infected individuals (Nikitin et al., 2014). In particular, influenza virus particles circulate in the air; the higher the airborne virus concentrations, the higher is the influenza infection risk among humans. Nevertheless, on exposure to low virus particle concentrations, the immune system can aid healthy individuals in resisting influenza virus infections (Hall, 2007; Nikitin et al., 2014; Tellier, 2009). Influenza is a global occurrence, with estimated annual incidence rates of 5–10% in adults and 20–30% in children. A common complication of influenza is secondary bacterial pneumonia, particularly prevalent among older adults and individuals with chronic diseases, which can lead to further elevations in morbidity and mortality rates. The frequent recurrence of influenza (Behzadinasab et al., 2020) indicates the significance of infectious disease prevention (WHO, 2024c).

The airborne transmission of SARS-CoV-2 and influenza viruses is influenced by various factors, such as temperature, humidity, and solar ultraviolet (UV) radiation (Bandara et al., 2023). In addition to direct person-to-person transmission, these viruses can be transmitted indirectly through contact with contaminated surfaces. Although high temperature, low pH, or high salinity conditions may reduce virus stability, the potential for contact transmission via the skin should be considered (Brown et al., 2009; Kratzel et al., 2020; Paek et al., 2010; Sun et al., 2020). Thus, a comprehensive assessment of the stability and survival of SARS-CoV-2 and influenza viruses on various surfaces, along with the efficacy of disinfectants on these surfaces, is essential for evaluating the risk of contact transmission and formulating effective infection control strategies (Hirose et al., 2020). Herein, we systematically review pertinent research on the environmental stability of SARS-CoV-2 and influenza viruses, examine the efficacy of various disinfectants against them, and provide evidence that may aid in protecting relevant personnel and guide future efforts to reduce the potential for virus spread and control.

## 2 SARS-COV-2 and influenza virus detection on contaminated surfaces of objects in various environments

### 2.1 Viral contamination in clinical environments

**Figure 1:**
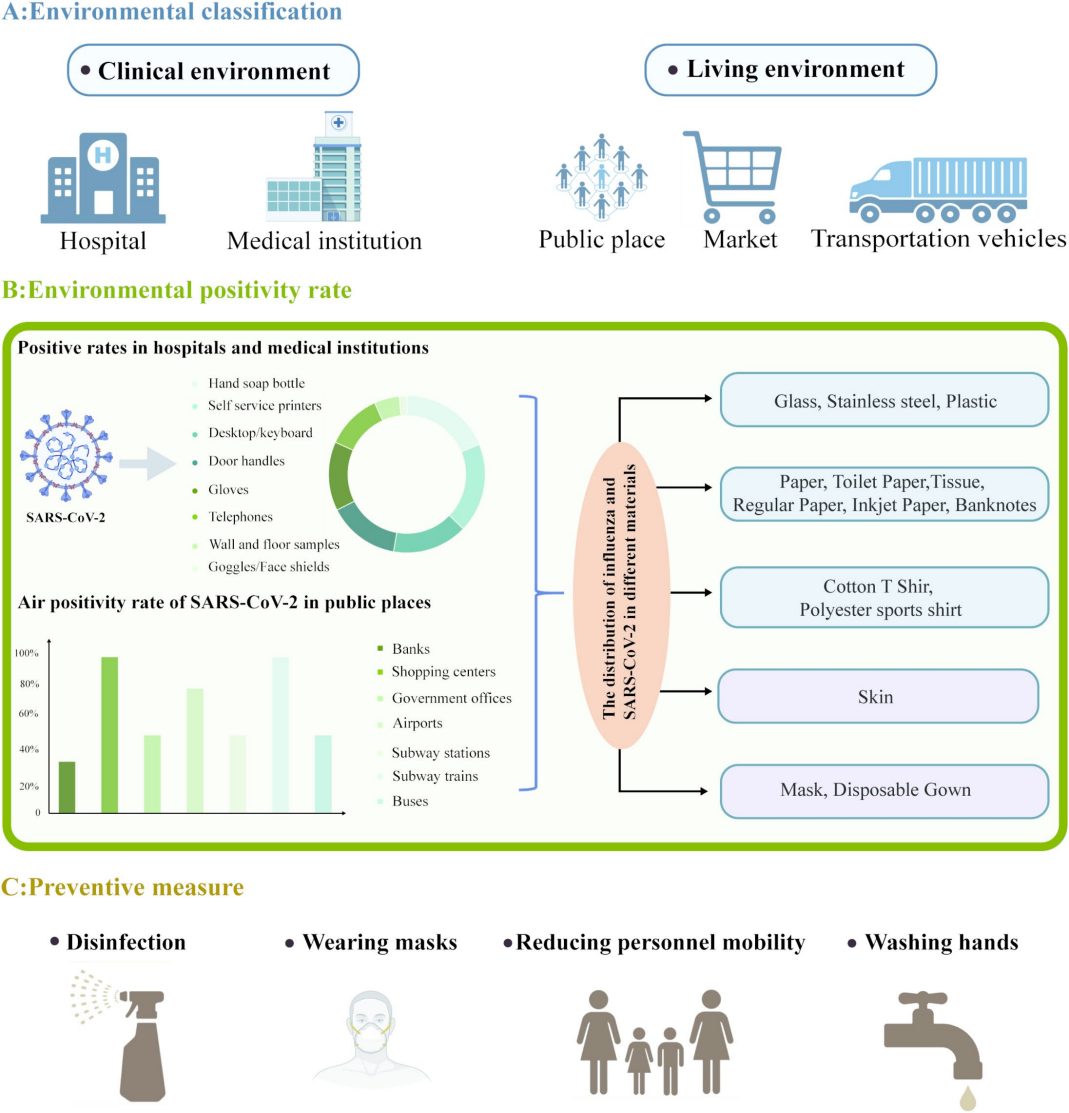
Overview of SARS-CoV-2 environmental distribution and preventive measures. (A) SARS-CoV-2 has been detected in clinical and living environments. (B) Showcased the positivity rates and pie charts of SARS-CoV-2 commonly used hospital items and medical equipment in hospitals and medical institutions; SARS-CoV-2 air positivity rate and bar chart in public places; The distribution of influenza and SARS-CoV-2 in different materials. (C) Multiple preventive measures. Created with https://app.biorender.com/gallery/illustrations.

In the healthcare sector, viral contamination in clinical settings remains a major concern. However, this contamination extends beyond medical institutions, encompassing crowded public spaces such as schools, public transport, and shopping centers, harboring potential risks of virus transmission (Cai et al., 2020; Donohue and Miller, 2020; Luo et al., 2020) (see Figure 1). This concern is particularly amplified during outbreaks, such as the COVID-19 pandemic and periods of heightened seasonal influenza virus activity, indicating the importance of understanding and managing viral contamination in the environment. Consequently, gaining insights into viral contamination in clinical settings can aid in devising effective preventive and control strategies for COVID-19 and influenza control. In this section, we explore how SARS-CoV-2 and influenza viruses are detected in clinical environments.

In healthcare facilities. SARS-CoV-2 has been discovered on surfaces and in air to which patients and healthcare workers are exposed. During the peak of the COVID-19 pandemic, from July 2020 to March 2021, Oksanen et al. (2022) collected 258 air and surface samples from hospitals and households. Moreover, Nagle et al. (2022) used reverse transcription quantitative polymerase chain reaction to assess the presence of SARS-CoV-2 RNA on various surfaces, implements, and air, excluding those in the intensive care unit, at Avicenne University Hospital, Assistance Publique-Hôpitaux de Paris, France, from January 22 to April 8, 2021. The authors detected SARS-CoV-2 RNA on surfaces (34%), air (12%), patient masks (50%), and healthcare workers’ masks (10%). Similarly, Ye et al. (2020) detected SARS-CoV-2 RNA on 13.9% of all the tested commonly used hospital items and medical equipment. The contaminated objects mainly included hand sanitizer bottles (20.3%), self-service printers (20.0%), desktop computers and keyboards (16.8%), door handles (16.0%), gloves (15.4%), telephones (12.5%), walls and floors (5.6%), and goggles and face shields (1.7%) (see Figure 1).

Regarding influenza, Mese et al. (2016) conducted a single-blind, cross-sectional study at nine different family medical centers in Istanbul, Türkiye. The study included a total of 238 participants. Among these individuals, 72 (30%) were younger than 19 years, classifying them into the pediatric group. The mean age of adult participants was 42.4 years, while the average age for children was 10.2 years. Out of the 238 patients, 122 were found to be positive for influenza RNA. The Veritor™ (BD Veritor) system demonstrated clinical sensitivity and specificity rates of 80 and 94%, respectively, across all age groups. Additionally, the positive predictive value was 93%, and the negative predictive value was 81% A. Chamseddine et al. (2021) conducted a study in which they collected 51 air samples from the rooms of patients diagnosed with influenza. The findings revealed that 51% of these samples were positive for the influenza A virus (IAV) RNA. Among the patients who tested positive for IAV, 65% were classified as emitters (defined as having at least one positive air sample), indicating a notably higher risk of in-hospital transmission in comparison to non-emitters. However, objective data available on influenza virus contamination rates in clinical settings are relatively limited.

Taken together, these findings highlight SARS-CoV-2 transmission risks in healthcare settings, underscoring the need for efficient management and cleaning of all surfaces and air in hospitals and clinics. These results have major implications for devising strategies that ensure safety in various medical environments.

### 2.2 Viral contamination in community environments and objects

In late 2019, a cluster of patients with unexplained pneumonia was linked to the South China Seafood Wholesale Market in Wuhan, Hubei, China. This market primarily trades in fruits, vegetables, seafood, and wild animals including hedgehogs (Li et al., 2020; World Health Organization, 2020). In June 2020, 335 confirmed cases were discovered at the Xinfadi Wholesale Market in Beijing, China; environmental samples and samples obtained from chopping boards used for imported salmon on the market were also positive for SARS-CoV-2 RNA (Li S. et al., 2022; Caiyu, 2020). In July 2020, SARS-CoV-2 (live) was detected on the surfaces of cold chain food packaging and containers in cities such as Dalian and Qingdao in China, as well as in various parts of South America (Liu et al., 2020). On August 12, 2020, local authorities in Shenzhen, Guangdong, China, detected nucleic acids on the packaging materials of frozen shrimp and surfaces of frozen chicken wings imported from Brazil, as well as inside their shipping containers; this was the first instance of SARS-CoV-2 detection in food samples (Han et al., 2021). This series of events indicates that SARS-CoV-2 can survive in community environments, increasing COVID-19 transmission risk and highlighting the need to assess potential sources of infection in the food supply chain and implement environmental hygiene and food safety measures.

Since the onset of the COVID-19 outbreak, SARS-CoV-2 nucleic acid has been detected in residential areas and public spaces. In Tehran, Iran, researchers analyzed the air in various public places between June and July 2020. They detected SARS-CoV-2 RNA in 64% of the samples, with the positivity rates being 62 and 67% in public places and transportation, respectively. SARS-CoV-2-positive samples were obtained from various locations such as banks (33%), shopping malls (100%), government offices (50%), airports (80%), subway stations (50%), subway trains (100%), and buses (50%) (Hadei et al., 2021) (see Figure 1). Guadalupe et al. (2021) assessed the presence of SARS-CoV-2 RNA on environmental surfaces during the outbreak and reported positivity rates of 11.11% (2/18), 10.17% (18/177), and 9.52% (8/84) on samples collected from wood, metal, and plastic surfaces, respectively. In contrast, the authors detected no viral RNA on glass (0/17) and ceramic (0/4) surfaces. Thus, wood, metal, and plastic surfaces may be relatively more prone to retaining SARS-CoV-2 RNA. SARS-CoV-2 has been reported to be more stable on plastic and stainless steel surfaces, with viable particles being detectable even after 21–28 days of contact (Guadalupe et al., 2021; Suman et al., 2020; van Doremalen et al., 2020). Although glass and ceramic surfaces tested negative for SARS-CoV-2 RNA, these results may be biased due to sample size variations among different surfaces; as such, the potential involvement of these surfaces in transmitting COVID-19 cannot be ruled out (Guadalupe et al., 2021).

He et al. (2015) monitored 1,488 poultry-related sites in Zhejiang, China, from March 2013 to February 2015. Positivity for the RNA of the H7N9 influenza virus did not differ significantly between urban and rural areas of the towns (33.7 and 31.0%, respectively; *p* = 0.543), sites (15.4 and 16.3%, respectively; *p* = 0.711), and environmental specimens (5.9 and 6.0%, respectively; *p* = 0.730). Furthermore, among the poultry-related sites, the H7N9 RNA was detected in drinking water samples (4.3%, 63/1482), sewage from poultry cleaning (8.6%, 105/1228), swabs from tables used for slaughtering or processing poultry (9.8%, 172/1760), and live poultry markets (8.8%, 785/8966). Moreover, 34 (3.7%) of the recruited 912 poultry-related workers tested positive for H7N9 antibodies.

These findings indicate that SARS-CoV-2 and influenza viruses can be detected (nucleic acid or infectious virus) in both community settings and on various surfaces, highlighting the need for efficient management and sanitation practices within these regions. Thus, to effectively mitigate the transmission of the epidemic caused by these viruses, a range of measures—including minimizing human traffic in public spaces, intensifying disinfection protocols for vehicles and food packaging surfaces, and enforcing mandatory mask usage—must be implemented (Liu et al., 2020).

## 3 SARS-COV-2 and influenza virus stability on environmental surfaces

### 3.1 Stability of SARS-CoV-2 and influenza viruses in varied environmental conditions

SARS-CoV-2 and influenza viruses remain the two major pathogen types globally. SARS-CoV-2 has spread worldwide and influenza viruses impose substantial health and economic burdens annually (de Francisco Shapovalova et al., 2015; Simonsen, 1999). In this section, we explore research findings concerning the survival of SARS-CoV-2 and influenza viruses under varied environmental conditions, including temperature, pH, and titer, and assess the importance and relevance of these findings in addressing pandemic-level challenges.

In their study on the thermal stability of SARS-CoV-2, Chin et al. investigated the virus’s stability at various temperatures using a concentration of 10^6.8^ 50% tissue culture infectious dose (TCID50)/mL (Chin et al., 2020). Their findings reveal that within the culture supernatant, the virus remained stable for up to 14 days at 4°C but survived for only 7 days at 22°C. At 70°C, the virus’s survival time decreased to only 5 min. Thus, SARS-CoV-2 retains infectious potential at cooler temperatures, whereas it is considerably vulnerable to heat (Chin et al., 2020). Subsequently, the authors analyzed the stability of SARS-CoV-2 (10^6.8^ TCID50/mL) further at room temperature across various pH levels. Exposure of virus-containing surfaces to the various conditions for 1 h led to the virus concentration decreasing to 10^5.51–^10^5.75^ TCID50/mL within a pH range of 3–10, demonstrating that SARS-CoV-2 is relatively stable under weakly acidic to alkaline environments (Chin et al., 2020). The authors measured SARS-CoV-2 survival rate of 1.2 × 10^3^ plaque-forming units (PFU) of the virus after a 30-s treatment in acidic saline (pH 2.2) at 60 min, which did not significantly affect the survival rate. The authors also reported that 1.2 × 10^3^ PFU of SARS-CoV-2 could survive for 3–4 days in a liquid medium or on a dry filter paper (Mese et al., 2016; Sun et al., 2020). This result further emphasizes that SARS-CoV-2 is relatively stable in a moist or dry environment, highlighting the importance of using disinfectants for sterilization and maintaining hand hygiene.

Poulson et al. (2016) investigated the stability of six human influenza A virus (IAV) strains, including pandemic (H1N1) and nonpandemic strains, under various environmental conditions, including various temperatures, pH, and salinity, with the virus concentrations ranging between 10^6.5^ and 10^7.9^ TCID50/mL. This research provided critical insights into IAV’s adaptability to different environments. The authors initially assessed the stability of all IAV strains in aqueous solutions at a neutral (7.2) pH across a range of temperatures and noted that all six strains demonstrated a time to 90% reduction in concentration (Rt) of 30–160 days at 4°C and that of only 0.9–4 days at 37°C. Notably, all strains demonstrated similar levels of environmental resilience, suggesting that they exhibit similar stability under similar conditions. Further analysis indicated that all strains were the most stable at 17°C and a pH of 7.2, with an Rt of 45 days. In contrast, a decrease in pH to 5.8 significantly shortened the Rt to <5 days on average. Finally, the authors investigated the impact of salinity on all six IAV strains and noted that at 17°C and a pH of 7.2, salinity led to a detrimental effect on the strains. In particular, at 0-ppt salinity in freshwater, all strains exhibited the highest Rt. As the salinity increased from 0 to 5 ppt, IAV stability declined rapidly, with an Rt of approximately 15 days.

Taken together, these results not only provide a new perspective on the survival mechanisms of SARS-CoV-2 and influenza viruses but also provide scientific bases for formulating the corresponding prevention and control strategies. Nevertheless, future studies should explore virus–environment interactions further.

### 3.2 SARS-CoV-2 stability on material surfaces

A series of studies have focused on SARS-CoV-2 stability across a diverse array of materials, including stainless steel, plastic, glass, various paper types (e.g., toilet paper, paper towels, printing paper, and banknotes), fabric, and wood, along with numerous types of protective equipment (Table 1). Chin et al. (2020) conducted one of the first studies evaluating the survivability of SARS-CoV-2 under various environmental conditions on substrates such as paper, toilet paper, wood, fabric, glass, banknotes, stainless steel, plastic, and face masks (inner and outer layers). Their findings revealed that at 22°C and 65% relative humidity, SARS-CoV-2 demonstrated differing survival durations on various materials, including glass and banknotes (2–4 days) and stainless steel and plastic (4–7 days). In contrast, the virus persisted for only 0.5–3 h on paper and toilet paper and 1–2 days on wood and fabric. These results suggest that surfaces of some materials, such as stainless steel and plastic, are potential vectors for SARS-CoV-2 transmission. Chin et al. (2020) also assessed SARS-CoV-2 survival on various material surfaces under different temperatures and reported that at 4°C, the infectious titer of the virus decreased by only approximately 0.7 log units after 14 days. However, when the incubation temperature was increased to 70°C, virus inactivation occurred within 5 min. Therefore, low-temperature conditions favor virus survival on various material surfaces, whereas high-temperature conditions enable significant and rapid virus inactivation on these surfaces (Tables 1–3). In another study, Chin et al. (2022) analyzed the survival characteristics of SARS-CoV-2, both the ancestral and Omicron BA.1 variants, on the surfaces of tissues, printing paper, plastic, glass, and stainless steel at 21°C–22°C. Compared with ancestral SARS-CoV-2, the Omicron BA.1 variant was more stable on each of the tested materials. Infectious ancestral SARS-CoV-2 was not detected on tissue and printing paper within 30 min of inoculation. Moreover, no infectious ancestral SARS-CoV-2 was recovered from the surfaces of plastic, glass, and stainless steel, even 7 days after inoculation. In contrast, the Omicron BA.1 variant remained detectable on the surfaces of plastic, glass, and stainless steel on the 7th inoculation day. These results suggest that the Omicron BA.1 variant has longer survival on these durable materials than ancestral SARS-CoV-2, and this enhanced survivability may increase the risk of transmission of the Omicron BA.1 variant (Chin et al., 2022; Eggink et al., 2022).

**Table 1:** SARS-CoV-2 stability on material surfaces.

Material	Strain	Temperature (°C)	Relative humidity (%)	Virus titer	Survival time	Half-life (95% CI)	Reference
Paper	\	22	65	3.16 × 10^5^ TCID_50_	0.5-3 h	\	Chin et al. (2020)
Tissue paper	\	22	65	3.16 × 10^5^ TCID_50_	0.5-3 h	\	Chin et al. (2020)
Tissue paper	Ancestral (A)	22	\	\	15-30 min	\	Chin et al. (2022)
Tissue paper	Omicron (BA.1)	22	\	\	30-60 min	\	Chin et al. (2022)
Printer paper	Ancestral (A)	22	\	\	5-10 min	\	Chin et al. (2022)
Printer paper	Omicron (BA.1)	22	\	\	30-60 min	\	Chin et al. (2022)
Plain paper	Ancestral (A)	25	40–50	2.00 × 10^5^ TCID_50_	59.78 h	2.03 h* (1.82–2.27)	Hirose et al. (2022a)
Inkjet paper	Ancestral (A)	25	40–50	2.00 × 10^5^ TCID_50_	6.48 h	0.22 h* (0.15–0.33)	Hirose et al. (2022b)
Inkjet photo paper	Ancestral (A)	25	40–50	2.00 × 10^5^ TCID_50_	9.78 h	0.34 h* (0.24–0.51)	Hirose et al. (2022a)
US$20 bills	Ancestral (A)	37	50	3.20 × 10^4^TCID_50_	8-24 h	0.2–0.6 h	Baker and Gibson (2022)
Banknotes	Ancestral (A)	21.5	45	4.00 × 10^5^ TCID_50_	5d	\	Paton et al. (2021)
Banknotes	\	22	65	3.16 × 10^5^ TCID_50_	2-4d	0.9 h	Chin et al. (2020)
Plastic	\	22	65	3.16 × 10^5^ TCID_50_	4-7d	1.6 h	Chin et al. (2020)
Plastic	Ancestral (A)	22	\	\	2-4d	\	Chin et al. (2022)
Plastic	Omicron (BA.1)	22	\	\	>7d	\	Chin et al. (2022)
Glass	\	22	65	3.16 × 10^5^ TCID_50_	2-4d	1.2 h	Chin et al. (2020)
Glass	Ancestral (A)	22	\	\	4-7d	\	Chin et al. (2022)
Glass	Omicron (BA.1)	22	\	\	>7d	\	Chin et al. (2022)
Stainless steel	\	22	65	3.16 × 10^5^ TCID_50_	4-7d	0.3 h	Chin et al. (2020)
Stainless steel	Ancestral (A)	22	\	\	2-4d	\	Chin et al. (2022)
Stainless steel	Omicron (BA.1)	22	\	\	>7d	\	Chin et al. (2022)
Stainless steel	Ancestral (A)	21.5	45	4.00 × 10^5^ TCID_50_	7d	\	Paton et al. (2021)
Stainless steel (DMEM)	Ancestral (A)	25	45–55	1.00 × 10^5^ TCID_50_	84.29 h	32.62 h (16.80–56.68)	Hirose et al. (2020)
Glass and stainless steel	Ancestral (A)	22	60–70	3.00× 10^5^ TCID_50_	>1d	\	Behzadinasab et al. (2020)
Stainless steel, polymer notes, paper notes, glass, and vinyl	Ancestral (A)	20	50	3.38× 10^7^ TCID_50_	>28d	1.8d, 2.06d, 2.74d, 1.90d, 1.91d	Riddell et al. (2020)
Stainless steel, polymer notes, and glass	Ancestral (A)	30	50	3.38× 10^7^ TCID_50_	>7d	12.6 h, 14,7 h, 10.5 h,	Riddell et al. (2020)
Stainless steel, polymer notes, paper notes, glass, cotton, and vinyl	Ancestral (A)	40	50	3.38× 10^7^ TCID_50_	1\2d	1.5 h, 1.4 h, 1.6 h, 2.0 h, \, 3.0 h	Riddell et al. (2020)
Cotton	Ancestral (A)	20	50	3.38× 10^7^ TCID_50_	7\14d	1.68d	Riddell et al. (2020)
Cotton	Ancestral (A)	30	50	3.38× 10^7^ TCID_50_	2\3d	11.0 h	Riddell et al. (2020)
Mask, outer layer	\	22	65	\	>7d	1.4 h	Chin et al. (2020)
Mask, inner layer	\	22	65	\	4-7d	1.0 h	Chin et al. (2020)
Surgical mask	Ancestral (A)	21.5	45	\	>7d	\	Paton et al. (2021)
Wood	\	22	65	\	1-2d	\	Chin et al. (2020)
Cloth	Ancestral (A)	22	65	\	1-2d	\	Chin et al. (2020)
Disposable gown	Ancestral (A)	21.5	45	4.00 × 10^5^ TCID_50_	7d	\	Paton et al. (2021)
Polyester sports shirt	Ancestral (A)	21.5	45	4.00 × 10^5^ TCID_50_	1d	\	Paton et al. (2021)
Cotton t-shirt	Ancestral (A)	21.5	45	4.00 × 10^5^ TCID_50_	5d	\	Paton et al. (2021)
Borosilicate glass (DMEM)	Ancestral (A)	25°C	45–55	1.00 × 10^5^TCID_50_	85.74 h	33.24 h (17.59–56.49)	Hirose et al. (2020)

*The concentration titer during half-life detection is 1.00 × 10^4^ TCID50.

**Table 2:** Survival of SARS-CoV-2 and influenza viruses on the skin surface.

Material	Strain name	Temperature (°C)	Relative humidity (%)	Virus titer	Survival time	Reference
Skin (Swine)	SARS-CoV-2	4	50	1.00 × 10^4.5^TCID_50_	>336 h	Baker and Gibson (2022) and Harbourt et al. (2020)
Skin (Swine)	SARS-CoV-2	22	50	1.00 × 10^4.5^TCID_50_	96-168 h	Baker and Gibson (2022) and Harbourt et al. (2020)
Skin (Swine)	SARS-CoV-2	37	50	1.00 × 10^4.5^TCID_50_	8-24 h	Baker and Gibson (2022) and Harbourt et al. (2020)
Skin (Human, DMEM)	SARS-CoV-2	25	45–55	1.00 × 10^4.5^TCID_50_	9.04 h	Hirose et al. (2020)
Skin (Human, Mucus)	SARS-CoV-2	25	45–55	1.00 × 10^4.5^TCID_50_	11.09 h	Hirose et al. (2020)
Skin (Human, DMEM)	H1N1	25	45–55	1.00 × 10^4.5^TCID_50_	1.82 h	Hirose et al. (2020)
Skin (Human, Mucus)	H1N1	25	45–55	1.00 × 10^4.5^TCID_50_	1.69 h	Hirose et al. (2020)
Skin (Human)	H5N1	25	45–55	4.00 × 10^5^TCID_50_	4.5 h	
Skin (Human)	H7N9, H5N3, H5N9, H3N2, H1N1	25	45–55	4.00 × 10^5^TCID_50_	2 h	Bandou et al. (2022)

**Table 3:** Differences in environmental stability among different SARS-CoV-2 variants.

Material	Strain	Temperature (°C)	Relative humidity (%)	Virus titer	Survival time	Half-life (95% CI)	Reference
Plastic	Wuhan strain (A)	25	45–55	5.01 × 10^4^TCID_50_	56 h	3.5 h* (2.0–5.7)	Hirose et al. (2022a)
Plastic	Alpha variant (B.1.1.7)	25	45–55	5.01 × 10^4^TCID_50_	191.3 h	9.9 h* (7.9–12.7)	Hirose et al. (2022b)
Plastic	Beta variant (B.1.351)	25	45–55	5.01 × 10^4^TCID_50_	156.6 h	8.3 h*(6.4–10.9)	Hirose et al. (2022a)
Plastic	Gamma variant (P.1)	25	45–55	5.01 × 10^4^TCID_50_	59.3 h	3.9 h* (2.5–5.8)	Hirose et al. (2022b)
Plastic	Delta variant (B.1.617.2)	25	45–55	5.01 × 10^4^TCID_50_	114 h	6.7 h *(5.1–8.7)	Hirose et al. (2022a)
Plastic	Omicron (BA.1)	25	45–55	5.01 × 10^4^TCID_50_	193.5 h	10.0 h* (8.0–12.9)	Hirose et al. (2022a)
Plastic	Omicron (BA.2)	25	45–55	5.01 × 10^4^TCID_50_	199.7 h	10.3 h* (8.4–12.7)	Hirose et al. (2022b)
Skin	Wuhan strain (A)	25	45–55	5.01 × 10^4^TCID_50_	8.6 h	0.5 h* (0.3–0.7)	Hirose et al. (2022a)
Skin	Alpha variant (B.1.1.7)	25	45–55	5.01 × 10^4^TCID_50_	19.6 h	1.1 h* (0.8–1.6)	Hirose et al. (2022b)
Skin	Beta variant (B.1.351)	25	45–55	5.01 × 10^4^TCID_50_	19.1 h	1.2 h*(0.8–1.8)	Hirose et al. (2022a)
Skin	Gamma variant (P.1)	25	45–55	5.01 × 10^4^TCID_50_	11 h	0.7 h* (0.5–1.1)	Hirose et al. (2022b)
Skin	Delta variant (B.1.617.2)	25	45–55	5.01 × 10^4^TCID_50_	16.8 h	1.0 h* (0.8–1.4)	Hirose et al. (2022a)
Skin	Omicron (BA.1)	25	45–55	5.01 × 10^4^TCID_50_	21.1 h	1.4 h* (1.0–2.0)	Hirose et al. (2022a)
Skin	Omicron (BA.2)	25	45–55	5.01 × 10^4^TCID_50_	22.5 h	1.3 h* (0.9–2.0)	Hirose et al. (2022b)
Paper carton	Omicron (BA.1)	4	40	1.00 × 10^3^TCID_50_	1-3d	\	Wang et al. (2023)
Paper carton	Omicron (BA.1)	25	40	1.00 × 10^3^TCID_50_	<6 h	\	Wang et al. (2023)
Paper carton	Omicron (BA.1)	37	40	1.00 × 10^3^TCID_50_	<6 h	\	Wang et al. (2023)
Paper carton	Omicron (BA.5)	4	40	1.00 × 10^3^TCID_50_	1-3d	\	Wang et al. (2023)
Paper carton	Omicron (BA.5)	25	40	1.00 × 10^3^TCID_50_	6-24 h	\	Wang et al. (2023)
Paper carton	Omicron (BA.5)	37	40	1.00 × 10^3^TCID_50_	<6 h	\	Wang et al. (2023)
PE packaging film	Omicron (BA.1)	4	40	1.00 × 10^3^TCID_50_	5-7d	\	Wang et al. (2023)
PE packaging film	Omicron (BA.1)	25	40	1.00 × 10^3^TCID_50_	1-3d	\	Wang et al. (2023)
PE packaging film	Omicron (BA.1)	37	40	1.00 × 10^3^TCID_50_	<6 h	\	Wang et al. (2023)
PE packaging film	Omicron (BA.5)	4	40	1.00 × 10^3^TCID_50_	>7d	\	Wang et al. (2023)
PE packaging film	Omicron (BA.5)	25	40	1.00 × 10^3^TCID_50_	1-3d	\	Wang et al. (2023)
PE packaging film	Omicron (BA.5)	37	40	1.00 × 10^3^TCID_50_	<6 h	\	Wang et al. (2023)

*The concentration titer during half-life detection is 1.00 × 10^3^ TCID50.

Hirose et al. (2022a) investigated SARS-CoV-2 persistence on plain paper (PP), inkjet paper (IP), and inkjet photo paper (IPP) at 25°C under a viral load of 2.0 × 10^5^ TCID50/mL and 40–50% relative humidity. Their results indicated that the virus persisted for 59.78, 6.48, and 9.78 h on PP, IP, and IPP, respectively, with SARS-CoV-2 exhibiting notably longer survival on PP than on IP and IPP. Hirose et al. also explored the drying duration of 2 μL of the culture medium Dulbecco’s modified Eagle’s medium (DMEM) and SARS-CoV-2-inactivation timeframe on these paper types. The findings revealed that DMEM dried considerably faster on IP and IPP than on PP; this difference is attributable to the surface treatments of IP and IPP, which enhance rapid drying by preventing ink seepage. Consequently, SARS-CoV-2 inactivation was more rapid on IP and IPP than on PP, leading to reduced virus survivability. Thus, IP and IPP may be considered safer for use in future epidemic mitigation efforts. Studies have also indicated that the virus remains viable on banknotes for ≥8 h at an elevated (37°C) temperature and typically for 2–5 days at 22°C. This result demonstrates that SARS-CoV-2 tends to be more stable on cooler, moister surfaces, whereas viral inactivation occurs more rapidly under drier conditions (Chin et al., 2020; Harbourt et al., 2020; Paton et al., 2021; Smither et al., 2020).

Viruses can remain stable on protective gear, including both masks and disposable gowns. In controlled environments at 22°C and 65% relative humidity, SARS-CoV-2 remains stable for 4 days on the inner layer of masks; this duration can extend up to 7 days on its outer layer (Chin et al., 2020; Paton et al., 2021). Similarly, at 22°C and only 45% relative humidity, disposable gowns can harbor the virus for ≥7 days (Paton et al., 2021).

These findings highlight the major influence of material composition and structure on viral stability. In the routine utilization of protective gear, the potential contamination hazards posed by masks and disposable gowns should be diligently addressed, with adherence to rigorous standards for their selection, use, and disposal. Moreover, SARS-CoV-2 survivability varies across different material surfaces (Table 1); this finding offers insights critical for deciphering the virus’s transmission dynamics and formulating relevant effective preventive measures. Notably, evidence from numerous studies has indicated the extended viability of SARS-CoV-2 on surfaces such as glass, plastic, paper, and stainless steel. It emphasizes the importance of intensifying disinfection protocols for these materials and considering material characteristics when selecting protective equipment so as to limit the spread of viruses in future pandemics (Gidari et al., 2021; Xu et al., 2023; Marquès and Domingo, 2021).

### 3.3 Influenza virus stability on material surfaces

In the investigation of transmission pathways of infectious disease, the comprehension of pathogen survivability and transmissibility on various material surfaces is essential. Influenza is a pervasive respiratory infection; understanding the related transmission dynamics and viral stability is critical to managing influenza outbreaks. In this section, we focus on the stability of influenza viruses across diverse material surfaces. Here, we summarize the results of studies methodically examining the effects of various material surfaces on influenza virus stability to bridge the existing knowledge gap and furnish a scientific foundation for influenza prevention and outbreak management.

Thompson and Bennett (2017) evaluated the survival of five IAV (H1N1) strains on different surfaces (cotton, microfiber, and stainless steel). At 19.5°C and 55.3% relative humidity, the time required to reduce the viral titers by 99% was 17.7, 34.3, and 174.9 h on cotton, microfiber, and stainless steel, respectively. Moreover, Hirose et al. (2022b) assessed the stability of IAV (H1N1) mixed with DMEM or upper respiratory tract mucus (hereafter, mucus) on material surfaces such as stainless steel, borosilicate glass, and polystyrene, and compared these results with those for SARS-CoV-2 (Table 4). The survival durations of the virus on stainless steel, borosilicate glass, and polystyrene were, respectively, 11.56, 10.61, 6.07, and 1.82 h when mixed with DMEM and 1.73, 1.73, and 1.96 h when mixed with mucus. Notably, IAV inactivation occurred faster in mucus than in DMEM. Moreover, SARS-CoV-2 was noted to survive approximately eight times longer on various material surfaces than IAV; as such, SARS-CoV-2 may be more stable in the environment than IAVs. McDevitt et al. (2010) assessed the survival duration of H1N1 under high-temperature, high-humidity conditions on stainless steel. At 65°C and 50 and 70% relative humidity, the influenza virus became inactivated within only 15 min. High temperatures can effectively inactivate influenza viruses. However, in practical operations, the inactivation time should be extended; because of the long time required for heating, the temperatures should also be increased as much as possible. This finding may facilitate practical applications of influenza virus inactivation.

**Table 4:** Influenza virus stability on material surfaces.

Material	Strain	Temperature (°C)	Relative humidity (%)	Virus titer	Survival time	Half-life (95% CI)	Reference
Plain paper	H3N2	25	40–50	2.00 × 10^5^ TCID_50_	10.29 h	0.62 h***(0.51–0.77)	Hirose et al. (2022a)
Inkjet paper	H3N2	25	40–50	2.00 × 10^5^ TCID_50_	1.75 h	0.11 h***(0.07–0.19)	Hirose et al. (2022b)
Inkjet photo paper	H3N2	25	40–50	2.00 × 10^5^ TCID_50_	3.32 h	0.20 h***(0.14–0.28)	Hirose et al. (2022a)
Cotton	H1N1	19.5	55.3	1.30 × 10^8^ TCID_50_	17.7 h	\	Thompson and Bennett (2017)
Cloth	H1N1	*	**	\	8 h	\	Oxford et al. (2014)
Plastic	H5N1	25	45-55	2.00 × 10^5^ TCID_50_	26 h	0.20 h*** (0.18–0.23)	Bandou et al. (2022)
Plastic	H7N9, H5N3, H5N9, H3N2, H1N1	25	45–55	2.00 × 10^5^ TCID_50_	<10 h	0.08 h***, 0.09 h***, 0.09 h***, 0.09 h***, 0.08 h***(0.08–0.10)	Bandou et al. (2022)
Formica (Plastic)	H1N1	\	\	2.94× 10^5^TCID_50_	60 min	\	Mukherjee et al. (2012)
Plastic	H1N1	*	**	\	24 h	\	Oxford et al. (2014)
Stainless steel	H1N1	19.5	55.3	1.30 × 10^8^ TCID_50_	174.9 h	\	Thompson and Bennett (2017)
Stainless steel	H1N1	\	\	2.94× 10^5^TCID_50_	15 min	\	Mukherjee et al. (2012)
Stainless steel	H1N1	25.2	55	1.00 × 10^3.8^TCID_50_	1d	\	Sakaguchi et al. (2010)
Stainless steel (DMEM)	H1N1	25	45–55	1.00 × 10^3.8^TCID_50_	11.56 h	6.78 h (5.84–7.97)	Hirose et al. (2020)
Stainless steel (Mucus)	H1N1	25	45–55	1.00 × 10^3.8^TCID_50_	1.73 h	\	Hirose et al. (2020)
Stainless steel	H1N1	55	75	1.60 × 10^5^TCID_50_	15 min	\	
Stainless steel	H1N1	60	50	1.60 × 10^5^TCID_50_	30 min	\	McDevitt et al. (2010)
Stainless steel	H1N1	65	50, 75	1.60 × 10^5^TCID_50_	15 min	\	
Stainless steel	H1N1	*	**	\	24 h	\	Oxford et al. (2014)
Stainless steel and plastic	H1N1	17–21	23–24	1.00 × 10 ^6^TCID_50_	<9 h	\	Greatorex et al. (2011)
Galvanized metal and plastic	H5N1	21.9–23.4	32–38	5.01 x 10^7^TCID_50_	1d	\	Wood et al. (2010)
Galvanized metal and plastic	H5N1	2.6–7.5	2.2–51.4	5.01 x 10^7^TCID_50_	>13d	\	Wood et al. (2010)
Surgical mask (nonwoven fabric)	H1N1	25.2	55	6.30 x 10^3^TCID_50_	1d	\	Sakaguchi et al. (2010)
Banknotes	H3N2	21–28	30–50	8.90 × 10^6^TCID_50_	3-4d	\	Thomas et al. (2008)
Banknotes	H3N2	21–28	30–50	5.00 × 10^4^TCID_50_	1-2d	\	Thomas et al. (2008)
Banknotes	H1N1	21–28	30–50	2.80 × 10^5^TCID_50_	1-2 h	\	Thomas et al. (2008)
Banknotes	Yamagata	21–28	30–50	1.60 × 10^4^TCID_50_	2-3 h	\	Thomas et al. (2008)
Banknotes and respiratory secretions	H3N2	21–28	30–50	8.90 × 10^6^TCID_50_	17-20d	\	Thomas et al. (2008)
Banknotes and respiratory secretions	Yamagata	21–28	30–50	3.20 × 10^3^TCID_50_	1-2d	\	Thomas et al. (2008)
Coated wooden desk	H1N1	25.2	55%	6.30 x 10^3^TCID_50_	1d	\	Sakaguchi et al. (2010)
Wood	H1N1	*	**	\	48 h	\	Oxford et al. (2014)
Topsoil	H5N1	22.0–23.9	30–42	5.01 × 10^6^–5.01 × 10^7^ TCID_50_	2d	\	Wood et al. (2010)
Facial tissue	H1N1	\	\	2.94 × 10^5^TCID_50_	15 min	\	Mukherjee et al. (2012)
Borosilicate glass (DMEM)	H1N1	25	45–55	1.00 × 10^5^TCID_50_	10.61 h	6.13 h (5.22–7.29)	Hirose et al. (2020)
Borosilicate glass (Mucus)	H1N1	25	45–55	1.00 × 10^5^TCID_50_	1.73 h	0.85 h (0.76–0.96)	Hirose et al. (2020)
Polystyrene (DMEM)	H1N1	25	45–55	1.00 × 10^5^TCID_50_	6.07 h	3.04 h (2.40–3.87)	Hirose et al. (2020)
Polystyrene (Mucus)	H1N1	25	45–55	1.00 × 10^5^TCID_50_	1.96 h	0.91 h (0.80–1.04)	Hirose et al. (2020)
Human skin (DMEM)	H1N1	25	45–55	1.00 × 10^5^TCID_50_	1.82 h	0.80 h (0.72–0.90)	Hirose et al. (2020)
Human skin (Mucus)	H1N1	25	45–55	1.00 × 10^5^TCID_50_	1.69 h	0.77 h (0.71–0.84)	Hirose et al. (2020)
Microfiber	H1N1	19.5	55.3	1.30 × 10^8^TCID_50_	34.3 h	\	Thompson and Bennett (2017)
Telephone handsets (plastic) and computer keyboards	H1N1	17–21	23–24	1.00 × 10^6^TCID_50_	<4 h	\	Greatorex et al. (2011)
Window glass, aluminum, pine (unsealed), and varnished and unvarnished oak	H1N1	17–21	23–24	1.00 × 10^6^TCID_50_	<4 h	\	Greatorex et al. (2011)
Silver-containing fabrics and soft toys	H1N1	17–21	23–24	1.00 × 10^6^TCID_50_	4-9 h	\	Greatorex et al. (2011)

*Home temperature; **household humidity; ***the concentration titer during half-life detection is 1.00 × 10^4^ TCID50.

Hirose et al. (2022a) discovered that under a specific set of conditions (viral load = 2.0 × 10^5^ focus-forming units (FFU), temperature = 25°C, and relative humidity = 40–50%), influenza virus H3N2 survived for 10.29, 1.75, and 3.32 h on PP, IP, and IPP, respectively. Moreover, the survival duration was considerably shorter on both IP and IPP than on PP. The authors also compared the stability of SARS-CoV-2 and IAVs on these paper surfaces and reported that the survival duration of SARS-CoV-2 was approximately sixfold that of IAVs, indicating that SARS-CoV-2 has higher environmental stability and potentially greater transmission risk. Thomas et al. (2008) assessed the longevity of influenza viruses H1N1, H3N2, and Type B on banknotes. Influenza viruses H1N1 and Type B survived for 1–3 h, whereas influenza virus H3N2 persisted for 1–4 days.

Bandou et al. (2022) reported that Avian IAV (H5N1) demonstrated notably higher stability and contact transmission risk compared with other IAV strains. At 25°C and under 45–55% relative humidity, each strain demonstrated a viral titer of 2.0 × 10^5^ FFU. On plastic surfaces, the survival durations of H7N9, H5N3, H5N9, H3N2, and H1N1 did not exceed 10 h; in contrast, H5N1 survived for a maximum of 26 h (Bandou et al., 2022). These findings highlight the superior stability of H5N1 on plastic surfaces and its high potential for contact transmission, indicating that this strain is a major public health concern.

In summary, influenza virus survival varies depending on material surface type, temperature and virus strain. This insight may aid in developing public health guidelines and strategies for influenza mitigation and control.

### 3.4 SARS-CoV-2 and influenza virus survival on skin surface

Contact transmission, particularly through direct human skin contact, is a major pathway for SARS-CoV-2 and influenza virus dissemination (Brankston et al., 2007). However, virus-infected skin is one of the typical sites for contact transmission (La Rosa et al., 2013; Louten, 2016). Therefore, evaluating the stability and survival of SARS-CoV-2 and influenza viruses on human skin is pivotal. This evaluation can not only increase the current understanding of potential risks associated with contact transmission but also provide crucial insights for development of additional efficacious infection control strategies (Hirose et al., 2020, 2021a). In addition to that on material surfaces, researchers have explored the survival duration of SARS-CoV-2 and influenza on skin surfaces (Table 2). Harbourt et al. (2020) revealed that at a viral titer of 4.5 log_10_PFU/mL, SARS-CoV-2 remained stable on porcine skin for ≥336, ≥96, and ≥ 8 h at 4°C, 22°C, and 37°C, respectively.

Bandou et al. (2022) investigated the stability of various influenza virus strains (4.0 × 10^5^ FFU) on human skin at 25°C with 45–55% relative humidity. The authors reported that all strains, except H5N1, survived for approximately 2 h (the time until virus on the surface is no longer detected). In contrast, H5N1 survived for approximately 4.5 h, which is 2.5 times longer than that of other strains. Moreover, H5N1 demonstrated a longer survival duration and stronger resistance to environmental conditions than other strains. As such, influenza virus H5N1 may be associated with a higher contact transmission risk than other influenza virus strains.

In summary, the survival duration of IAVs on the skin is shorter than that of SARS-CoV-2. Although the infectivity of IAVs decreases gradually over time, the viruses may pose a contact transmission risk for ≥2 h. Notably, in cold winter or similar low-temperature environments, SARS-CoV-2 demonstrates higher stability, extending its period of infectivity. Therefore, strict adherence to appropriate hand hygiene measures and social distancing guidelines is crucial during outbreaks of SARS-CoV-2 and influenza.

### 3.5 Differences in environmental stability among different SARS-CoV-2 variants

During the COVID-19 pandemic, several variants of concern of SARS-CoV-2 spread globally (Thye et al., 2021). The environmental stability of the Wuhan (ancestral) strain is higher compared to influenza viruses (Hirose et al., 2021b; Hirose et al., 2020). Considering its infectiousness, SARS-CoV-2 can not only be a major global public health concern but also inflict severe economic damage. For instance, previously emerged variants, such as B.1.1.7 and the subsequent Omicron variant, have presented significant public health challenges because of their high transmissibility (Bálint et al., 2022; Lyngse et al., 2021).

Hirose et al. (2022b) conducted compared the survival of SARS-CoV-2 variants, including the Wuhan strain (A), Alpha, Beta, Gamma, Delta, Omicron BA.1, and Omicron BA.2, on plastic and human skin surfaces under the following conditions: viral titer = 10^4.7^ TCID50, temperature = 25°C, and relative humidity = 45–55% (Table 3). Notably, the results demonstrated that the Alpha, Beta, Delta, and Omicron variants exhibited longer survival on both plastic and human skin surfaces—more than twice as long as the Wuhan strain (A). Notably, the survival durations of the Omicron BA.1 and BA.2 variants on plastic surfaces were 193.5 and 199.7 h, respectively, confirming that they had environmental stability similar to, or even higher than, that reported previously. Similarly, Wang et al. (2023) compared the environmental stability of Omicron BA.1 and BA.5 on different packaging materials under the following conditions: viral titer = 10^3^ TCID50, relative humidity = 40%, and temperature = 25°C. The results demonstrated that the Omicron BA.1 variant exhibited shorter survival than the Omicron BA.5 variant (≤6 vs. 6–24 h; Table 3). However, on box surfaces at 4°C and under 40% relative humidity and protectively equipment packaging material use, the Omicron BA.1 variant survived for 5–7 days, whereas the Omicron BA.5 variant survived for ≥7 days. As such, the Omicron BA.5 variant has slightly higher environmental stability than the Omicron BA.1 variant; however, variations in initial viral titers may have influenced these results. Therefore, Omicron strains of SARS-CoV-2 may be associated with an increase in transmission risks (Bálint et al., 2022; Ji et al., 2022).

## 4 Decontamination and disinfection of SARS-CoV-2 and influenza viruses in the environment

### 4.1 *In vitro* surface inactivation of SARS-CoV-2 and influenza viruses

Before the reports concerning SARS-CoV-2 inactivation emerged, Kampf et al. (2020) conducted the first in-depth examination of the effects of disinfectants on SARScoronavirus 1, SARS-CoV-2, and endemic human coronavirus. Their research revealed that, within 1 min, 62–71% ethanol, 0.5% hydrogen peroxide, or 0.1% sodium hypochlorite effectively inactived these viruses. However, other biocides, 0.05–0.2% benzalkonium chloride (BAC) or 0.02% chlorhexidine gluconate (CHG), demonstrated relatively inadequate efficacy (Kampf et al., 2020). As such, various disinfectants demonstrate varying coronavirus inactivation effects; this inference may aid in selecting appropriate prevention and control strategies.

With the emergence of the COVID-19 pandemic, research focus progressively shifted toward assessing the efficacy of disinfectants against various viruses, particularly SARS-CoV-2 and influenza viruses. Most studies were aimed at both addressing the pandemic and assessing the potential applicability of the disinfectants to a wide variety of respiratory viruses, including influenza viruses, so as to formulate comprehensive epidemic prevention strategies with broad applicability. In this section, we review the efficacy of common disinfectants for inactivating SARS-CoV-2 and influenza viruses (Table 5).

**Table 5:** Inactivation of SARS-CoV-2 and influenza viruses using different disinfectants on material surfaces.

Environment	Virus	Disinfectant	Concentration (%)	Action time	Supplier	Country or region	Virus titer decrease (log_10_TCID_50_) or percentage (%)	References
\	SARS-CoV-2	EA	75	1 min	VWR Chemicals BDH	USA	≥1.83 ± 0.29	Chan et al. (2020)
\	SARS-CoV-2	EA	75	5 min	VWR Chemicals BDH	USA	≥2.00 ± 0.29	Chan et al. (2020)
\	SARS-CoV-2	H2O2 3.0%	1.5	15 s	United States Pharmacopeia	USA	1.33	Bidra et al. (2020)
\	SARS-CoV-2	H2O2 6.0%	3	15 s	United States Pharmacopeia	USA	1.00	Bidra et al. (2020)
\	SARS-CoV-2	Bleach	10	1 min	Kao	Japan	≥3.25 ± 0.00	Chan et al. (2020)
\	SARS-CoV-2	Hand wash	-	1 min	Walch	Germany	≥0.83 ± 0.29	Chan et al. (2020)
\	SARS-CoV-2	Hand wash	-	5 min	Walch	Germany	≥0.92 ± 0.14	Chan et al. (2020)
\	SARS-CoV-2	Advanced hand sanitizer	-	1 min	Purell	USA	≥2.50 ± 0.0	Chan et al. (2020)
\	SARS-CoV-2	Liquid hand soap (Funchem)	-	5 min	Funchem	HKSAR	≥2.50 ± 0.0	Chan et al. (2020)
\	SARS-CoV-2	Liquid hand soap (Funchem)	-	1 min	Funchem	HKSAR	≥2.00 ± 1.56	Chan et al. (2020)
\	SARS-CoV-2	Formalin	10	1 min	Thermo fisher	USA	≥1.25 ± 0.00	Chan et al. (2020)
*In Vitro*	SARS-CoV-2	EA	40, 60, 80	5 s	Nacalai Tesque	Kyoto	>4.50	Hirose et al. (2021a)
*In Vitro*	SARS-CoV-2	EA	20	60s	Nacalai Tesque	Kyoto	0.33 ± 0.14	Hirose et al. (2021a)
*In Vitro*	SARS-CoV-2	IPA	70	5 s	Nacalai Tesque	Kyoto	>4.50	Hirose et al. (2021a)
*In Vitro*	SARS-CoV-2	CHG	0.2; 1	60s	Saraya	Kyoto	0.58 ± 0.14; 1.83 ± 0.29	Hirose et al. (2021a)
*In Vitro*	SARS-CoV-2	BAC	0.05; 0.2	60s	Yakuhan Pharmaceutical	Japan	2.17 ± 0.29; 3.00 ± 0.43	Hirose et al. (2021a)
\	SARS-CoV-2	PVP-I 1.0% Oral Rinse	1	15 s	Veloce BioPharma	Fort Lauderdale	>4.33	Bidra et al. (2020)
\	SARS-CoV-2	PVP-I 2.5% Oral Rinse	1.25	15 s	Veloce BioPharma	Fort Lauderdale	>4.33	Bidra et al. (2020)
\	SARS-CoV-2	PVP-I 3.0% Oral Rinse	1.5	15 s	Veloce BioPharma	Fort Lauderdale	>4.33	Bidra et al. (2020)
\	SARS-CoV-2	oral disinfectant PVP-I	1	1 min	\	\	>4.00	Wang et al. (2021)
\	SARS-CoV-2	oral disinfectant PVP-I	1	1 min	Veloce BioPharma	Fort Lauderdale	>4.00	Wang et al. (2021)
\	SARS-CoV-2	Hexadecyl Pyridine Chloride	0.1	2 min	\	\	>5.00	
*In Vitro*	H3N2	EA	40, 60, 80	5 s	Nacalai Tesque	Kyoto	>4.10	Hirose et al. (2021a)
*In Vitro*	H3N2	EA	20	60s	Nacalai Tesque	Kyoto	0.06 ± 0.07	Hirose et al. (2021a)
*In Vitro*	H5N1, H7N9, H5N3, H5N9, H3N2, H1N1	EA	40, 60, 80	15 s	Nacalai Tesque	Kyoto	>4.00	Bandou et al. (2022)
*In Vitro*	H7N9, H5N3, H5N9, H3N2, H1N1	EA	36	15 s	Nacalai Tesque	Kyoto	>4.00	Bandou et al. (2022)
*In Vitro*	H5N1-Ky, H5N1-Eg	EA	36	15 s	Nacalai Tesque	Kyoto	1.77–2.57	Bandou et al. (2022)
*In Vitro*	H7N9, H5N3, H5N9, H3N2, H1N1	EA	34	15 s	Nacalai Tesque	Kyoto	1.46–1.60	Bandou et al. (2022)
*In Vitro*	H5N1-Ky, H5N1-Eg	EA	34	15 s	Nacalai Tesque	Kyoto	0.28–0.29	Bandou et al. (2022)
*In Vitro*	H3N2	IPA	70	5 s	Nacalai Tesque	Kyoto	>4.10	Hirose et al. (2021a)
*In Vitro*	H5N1, H7N9, H5N3, H5N9, H3N2, H1N1	CHG	1.0	15 s	Saraya	Kyoto	1.05–1.59	Bandou et al. (2022)
*In Vitro*	H3N2	CHG	0.2; 1	60s	Saraya	Kyoto	0.19 ± 0.07; 0.40 ± 0.06	Hirose et al. (2021a)
*In Vitro*	H5N1, H7N9, H5N3, H5N9, H3N2, H1N1	BAC	0.2	15 s	Yakuhan Pharmaceutical	Japan	2.95–3.50	
*In Vitro*	H3N2	BAC	0.05; 0.2	60s	Yakuhan Pharmaceutical	Japan	2.71 ± 0.09; >4.07	Hirose et al. (2021a)

EA, ethanol; IPA, isopropanol; CHG, chlorhexidine gluconate; BAC, benzalkonium chloride; PVP-I, povidone-iodine.

The findings of Hirose et al. (2021a) and Bandou et al. (2022) concerning SARS-CoV-2 and various strains of influenza viruses merit attention. Hirose et al. (2021a) indicated that *in vitro* exposure to ethanol (40, 60%, or 80%) and isopropanol (70%) for >5 s can effectively inactive SARS-CoV-2 and influenza viruses (>4.1 log_10_TCID50). However, at a concentration of <40%, the SARS-CoV-2- and influenza virus-inactivating efficacy of ethanol diminishes significantly. For instance, 1-min exposure to 20% ethanol reduced SARS-CoV-2 and influenza virus titers by 0.33 ± 0.14 and 0.06 ± 0.07 log_10_TCID50, respectively; moreover, 15-s exposure to 34% ethanol reduced influenza virus titers by 1.46–1.60 log_10_TCID50. Bandou et al. (2022) reported that all influenza virus strains, except H5N1, were swiftly neutralized by 36% ethanol within 15 s, resulting in titer reductions of >4 log_10_TCID50. However, 36% ethanol demonstrated low inactivity efficacy against H5N1-Ky and H5N1-Eg, with titer reductions ranging from 1.77 to 2.57 log_10_TCID50. Therefore, compared with other strains, H5N1 may be more resistant to ethanol activity. This increases the risk of H5N1 transmission via contact relative to other influenza virus strains.

Hirose et al. (2021a) also focused on object surface disinfection and reported that >15-s exposure to 0.2% BAC demonstrated high SARS-CoV-2 disinfection efficacy (reduction by >2.96 log_10_TCID50). However, the effectiveness of 1% CHG remained suboptimal. In a study on reducing virus transmission during surgery, preoperative oral rinsing with 1% povidone-iodine (PVP-I) resulted in complete SARS-CoV-2 inactivation within only 15 s. Furthermore, in studies targeting oral hygiene, notable effectiveness was observed with disinfectants like PVP-I (1 mg/mL for 1 min) or cetylpyridinium chloride (0.1 mg/mL for 2 min). In a clinical trial, compared with CHG, PVP-I led to significant staining reduction and thus was preferred by patients (Fine, 1985). Moreover, at low concentrations, PVP-I demonstrates effective virucidal activity against SARS-CoV-2 and thus may aid in preventing future novel viral respiratory infections and offering new prospects for enhancing dental care (Eggers et al., 2018). In general, the aforementioned disinfectants can effectively inactivate SARS-CoV-2 and influenza viruses on the surfaces of various materials and could be part of future endeavors for combating transmission of emerging viruses.

### 4.2 Inactivation of SARS-CoV-2 and influenza virus (H3N2) on skin surface

SARS-CoV-2 and influenza virus H3N2 can be inactivated by disinfectants on human skin (Table 6). In particular, exposure to ethanol (35, 40, 60, 70%, or 80%) or isopropanol (70%) for >5 s considerably reduces the viral titers by >4.0 log_10_TCID50. However, 20% ethanol demonstrates considerably lower virus inactivation efficacy. At prescribed concentrations, ethanol can significantly mitigate activity of both SARS-CoV-2 and H3N2 (Zhu et al., 2020). Moreover, 1-min exposure to CHG (1%) or BAC (0.2%) can effectively neutralize these viruses (Chin et al., 2020). Exposure to substances such as PVP-I and hand sanitizers for >5 min also demonstrates effective SARS-CoV-2 and H3N2 inactivation. Thus, when used for disinfection of the skin, selecting appropriate concentrations of disinfectants and extending contact time accordingly is crucial.

**Table 6:** Inactivation of SARS-CoV-2 and influenza virus (H3N2) using disinfectants on skin surface.

Medium	Virus	Disinfectant	Concentration (%)	Action time	Supplier	Country or region	Virus titer decrease (log_10_TCID_50_) or percentage (%)	Reference
Skin (Human)	Wuhan strain (A), alpha variant (B.1.1.7), gamma variant (P.1), delta variant (B.1.617.2), omicron (BA.1), omicron (BA.2)	EA	35	15 s	Nacalai Tesque	Kyoto	>4.00	Hirose et al. (2022b)
Skin (Human)	Wuhan strain (A), alpha variant (B.1.1.7), gamma variant (P.1), delta variant (B.1.617.2), omicron (BA.1), omicron (BA.2)	EA	20	15 s	Nacalai Tesque	Kyoto	0.21–1.07	Hirose et al. (2022a)
Skin (Human)	SARS-CoV-2	EA	40, 60, 80	5 s	Nacalai Tesque	Kyoto	>4.19	Hirose et al. (2021a)
Skin (\)	SARS-CoV-2	EA	70	5 min			U	Chin et al. (2020)
Skin (Human)	SARS-CoV-2	IPA	70	5 s	Nacalai Tesque	Kyoto	>4.19	Hirose et al. (2021a)
Skin (Human)	SARS-CoV-2	CHG	0.2, 1	60s	Saraya	Kyoto	2.42 ± 0.18; 3.17 ± 0.33	Hirose et al. (2021a)
Skin (Human)	SARS-CoV-2	BAC	0.05, 0.2	60s	Yakuhan Pharmaceutical	Japan	2.36 ± 0.38; 3.19 ± 0.21	Hirose et al. (2021a)
Skin (\)	SARS-CoV-2	Household bleach	1	5 min	\	\	U	Chin et al. (2020)
Skin (\)	SARS-CoV-2	Hand soap solution	2	15 min	\	\	U	Chin et al. (2020)
Skin (\)	SARS-CoV-2	PVP-I	7.5	5 min	\	\	U	Chin et al. (2020)
Skin (\)	SARS-CoV-2	Chlorhexidine	0.05	5 min	\	\	U	Chin et al. (2020)
Skin (\)	SARS-CoV-2	BAC	0.1	5 min	\	\	U	Chin et al. (2020)
Skin (Human)	H3N2	EA	40, 60, 80	5 s	Nacalai Tesque	Kyoto	>4.12	Hirose et al. (2021a)
Skin (Human)	H3N2	IPA	70	5 s	Nacalai Tesque	Kyoto	>4.12	Hirose et al. (2021a)
Skin (Human)	H3N2	CHG	0.2, 1	60s	Saraya	Kyoto	1.02 ± 0.14; 3.39 ± 0.55	Hirose et al. (2021a)
Skin (Human)	H3N2	BAC	0.05, 0.2	60s	Yakuhan Pharmaceutical	Japan	1.23 ± 0.60; 3.24 ± 0.81	Hirose et al. (2021a)

EA, ethanol; IPA, isopropanol; CHG, chlorhexidine gluconate; BAC, benzalkonium chloride; PVP-I, povidone-iodine; U, undetectable.

### 4.3 Inactivation of SARS-CoV-2 and influenza viruses with ultraviolet radiation and ozone

Transmission of SARS-CoV-2 and influenza viruses is a major factor affecting global security and socioeconomic stability. Despite the advent of various preventive vaccines, environmental disinfection, along with personal protective measures, remains crucial during epidemic outbreaks. At present, numerous broad-spectrum disinfection strategies, including methods based on ultraviolet radiation and ozone, have emerged (She et al., 2020).

Ultraviolet radiation can be divided into short-wave (UVC; 200–280 nm), medium-wave (UVB; 280–320 nm), long-wave (UVA; 320–400 nm), and vacuum (UVD) types based on wavelength (Ploydaeng et al., 2021; Wang et al., 2013; Yin et al., 2013). Prolonged exposure to 254- and 275-nm ultraviolet radiation can cause harm to organic matter, as well as injure human and animal skin or eyes. Single irradiation with high doses of 222-nm UVC does not induce mutagenesis or cytotoxic DNA damage in mammalian cells (Narita et al., 2018). Consequently, 222-nm UVC is considered safer than 254- and 275-nm UVC.

Numerous studies have explored the impact of ultraviolet radiation on SARS-CoV-2 and influenza viruses (Table 7). Song et al. (2023) reported that ultraviolet radiation can effectively inactivate SARS-CoV-2 under 222-nm UVC (*d* = 50 mm, 2.5 mJ/cm^2^) or 275-nm UVC (*d* = 50 mm, 275 mJ/cm^2^) for 30 s, with a decrease in viral titer of >4.4 log_10_TCID50. Criscuolo et al. (2021) inactivated >94.4% of SARS-CoV-2 by irradiating surfaces of various materials, including glass, plastic, gauze, and wool, with 254-nm ultraviolet radiation (*d* = 200 mm, 1.8 mW/cm) for 15 min. Moreover, exposure to either 222-nm UVC (*d* = 300 mm, 48 mJ/cm^2^) for >10 min or UVB for 14 h (90 μW/cm) has been noted to effectively inactivate influenza viruses (Sutton et al., 2013; Welch et al., 2018; Xie et al., 2022).

**Table 7:** Inactivation of SARS-CoV-2 and influenza viruses using ultraviolet radiation, ozone, and other methods.

Medium	Virus	Disinfectant	Concentration (%)	Action time	Virus titer decrease (log_10_TCID_50_) or percentage (%)	Reference
Liquid	SARS-CoV-2	Ultraviolet Radiation (222 nm, *d* = 50 mm)	2.5 mJ/cm^2^/s	30s	4.40	Song et al. (2023)
Fabric	SARS-CoV-2	Ultraviolet Radiation (222 nm, *d* = 50 mm)	2.5 mJ/cm^2^/s	30s	4.72	Song et al. (2023)
Liquid	SARS-CoV-2	Ultraviolet Radiation (222 nm, *d* = 50 mm)	2.5 mJ/cm^2^/s	60s	U	Song et al. (2023)
Fabric	SARS-CoV-2	Ultraviolet Radiation (222 nm, *d* = 50 mm)	2.5 mJ/cm^2^/s	60s	U	Song et al. (2023)
Plastic, Stainless steel	SARS-CoV-2	Ultraviolet Radiation (254 nm)	20.06 mJ/cm^2^	\	≥4.00	Gidari et al. (2021)
Glass, plastic, gauze, and wool	SARS-CoV-2	UV-C (254 nm, *d* = 20 cm)	1.62 J/cm^2^	\	>99.9, >99.9, >99.9, 94.4%,	Criscuolo et al. (2021)
Liquid	SARS-CoV-2	Ultraviolet Radiation (275 nm, *d* = 50 mm)	275 mJ/cm^2^/s	10s	U	Song et al. (2023)
Fabric	SARS-CoV-2	Ultraviolet Radiation (275 nm, *d* = 50 mm)	275 mJ/cm^2^/s	10s	U	Song et al. (2023)
Glass	H1N1	UV-C (222 nm)	4.8 mJ/cm^2^/min	10 min	99.56%	Xie et al. (2022)
Glass	H3N2	UV-C (222 nm)	4.8 mJ/cm^2^/min	10 min	99.72%	Xie et al. (2022)
Steel	H1N1	UV-C (222 nm)	4.8 mJ/cm^2^/min	10 min	99.86%	Xie et al. (2022)
Steel	H3N2	UV-C (222 nm)	4.8 mJ/cm^2^/min	10 min	99.84%	Xie et al. (2022)
Air	H1N1	UV-C (222 nm)	2 mJ/cm^2^	\	>95%	Welch et al. (2018)
\	H3N2	UV-C (253.7 nm)	12.5 μW/cm^2^	3 min	>6.00	
Water	H5N1	UV-C (254 nm)	25, 40, 60 mJ/cm^2^	\	>5.50	Lénès et al. (2010)
Filtering facepiece respirator	H5N1	Ultraviolet Germicidal Irradiation	1.6–2.2 mW/cm^2^; 18 kJ/m^2^	15 min	>4.00	Lore et al. (2012)
Phosphate-buffered saline	H5N1	UV-B	90 μW/cm^2^	14 h	>5.20	Sutton et al. (2013)
Phosphate-buffered saline	H7N1	UV-B	90 μW/cm^2^	14 h	>5.50	Sutton et al. (2013)
Glass, plastic, gauze, wood, and wool	SARS-CoV-2	Ozone	0.2 ppm	120 min	90%; 82.2%; 96.8%; 93.3%;>99.9%	Criscuolo et al. (2021)
Glass, plastic, gauze, wood, and wool	SARS-CoV-2	Ozone	4 ppm	120 min	94.4%; 90%; 99.8%; 93.3%;99.7%	Criscuolo et al. (2021)
Plastic culture dish	H1N1	Ozone	20 ppm	150 min	4.00–5.00	Tanaka et al. (2009)
Plastic culture dish	H1N1	Ozone	10 ppm	210 min	4.00–5.00	Tanaka et al. (2009)
Water	H5N1, H1N1	Ozone	0.5, 1 mg/L	10 min	>4.00	Lénès et al. (2010)
Fetal bovine serum (0.5% or 5%)	H7N1	ClO2 (liquid state)	10 ppm	15 s	>4.58	Kadota et al. (2023)
Fetal bovine serum (0.5% or 5%)	IBV	ClO2 (liquid state)	10 ppm	15 s	>3.71	KADOTA et al. (2023)
Fetal bovine serum (0.5% or 5%)	IBV	ClO2 (Gaseous state)	1,500 to 2,000 ppb	3.5 min	>94.2%	Kadota et al. (2023)
Water	H5N1	Chlorine	Chlorine residual 0.3, 1, 1.5 mg/L	5 min	>4.00	Lénès et al. (2010)
Plastic microplates	H1N1	Dry fogging of hypochlorous acid solution	250 ppm	17 min	>3.00	Urushidani et al. (2022)
Plastic microplates	H1N1	Dry fogging of H2O2	11,280 ppm	17 min	>2.50	Urushidani et al. (2022)

U, undetectable.

Ozone is recognized by the WHO as a potent oxidant and one of the most effective disinfectants against microorganisms. It is extensively utilized for viral deactivation in aerosols (Clavo et al., 2020). Ozone can efficaciously inactive several pathogens including enteroviruses and mouse coronaviruses (Dubuis et al., 2020; Lin et al., 2007). Next, we will discuss the inactivation effect of ozone on SARS-CoV-2 and influenza virus in the air. Exposure to ozone at a lower concentration (0.2 ppm) for 30 min inactives 82.2–99.9% of SARS-CoV-2, whereas that at a higher concentration (4 ppm) for 120 min inactives 90–99.8% of the virus (Sutton et al., 2013). Variations in surface characteristics may, influence efficacy. Dubuis et al. (2021) demonstrated that subjecting influenza virus H1N1 to 1.70 ± 0.19 ppm ozone at 76% relative humidity for 80 min reduced the virus’s infectivity by four orders of magnitude. Moreover, exposure to 20 ppm ozone at 65% relative humidity for 150 min led to complete H1N1 deactivation, with a viral titer decrease of 4–5 log_10_TCID50. Similarly, Tanaka et al. (2009) reported that exposure to 20 ppm ozone at 65% relative humidity for 150 min completely inactivated H1N1, again resulting in a viral titer decrease of 4–5 log_10_TCID50 in viral titer. Notably, conventional gas-based chemical disinfectants such as chlorine dioxide and chlorine gas are effective against influenza viruses (Ibáñez-Cervantes et al., 2020; Lénès et al., 2010).

In summary, ultraviolet radiation (222, 254, or 275 nm), ozone, and chlorine dioxide can be effectively used for air disinfection against SARS-CoV-2 and influenza viruses.

## 5 Conclusion

Comprehensive research thus far has indicated that SARS-CoV-2 is more resilient and stable in the environment than influenza viruses. Under standard room temperature (20–25°C) and relative humidity (40–80%), the survival duration of both virus types on various surfaces is correlated with their titers: the higher the titer, the longer the survival. In particular, SARS-CoV-2 persists on the surface of materials such as stainless steel, plastic, and glass for 2–7 days; in contrast, under similar conditions, influenza viruses survive on stainless steel, plastic, and glass for 1–7 days, 1 day, and 1 day, respectively. On paper-based materials such as tissue paper, paper towels, and printing paper, SARS-CoV-2 persists for 1–3 h; in contrast, it persists for 2–3 days, 6 h, and 9 h on PP, IP, and IPP, respectively. Influenza viruses persist for 10, 1.75, and 3.32 h on PP, IP, and IPP, respectively. SARS-CoV-2 also survives on the outer layer of surgical masks for 7 days. Notably, SARS-CoV-2 and influenza viruses remain stable at lower temperatures (4°C), whereas they become inactive under acidic (pH < 2.2 for SARS-CoV-2; pH < 5.8 for influenza viruses) and high-temperature environments. Salinity also affects influenza viruses adversely. Both SARS-CoV-2 and influenza viruses exhibit relatively poor stability on human skin surfaces; nevertheless, they can survive for ≥2 h. Recent studies have suggested that the Omicron variant may demonstrate increased stability on material and skin surfaces, providing valuable insights for future epidemic control. As such, understanding the environmental stability and survival duration of both SARS-CoV-2 and influenza viruses, along with assessing the efficacy of disinfectants against these viruses on surfaces, is pivotal for formulating more effective infection control strategies.

In this review, we identified four primary limitations. First, influenza viruses remain prevalent in healthcare settings; however, detailed data regarding hospital contamination by influenza viruses are highly lacking. Influenza viruses exhibit transmission modes and characteristics different from those of SARS-CoV-2: SARS-CoV-2 infections consistently lead to high mortality because the mutations in the receptor-binding domain of SARS-CoV-2 enhance its transmissibility and lethality (Li C. et al., 2022). Consequently, researchers tend to prioritize the study of SARS-CoV-2 transmission and infectivity, and relatively few studies focus on influenza viruses in hospital environments. Therefore, future studies should focus on influenza virus contamination rates in hospitals to fill the aforementioned research gap and achieve a more comprehensive understanding of the behavioral characteristics of different viruses in the environment. Second, we primarily focused on early major variants of SARS-CoV-2, such as the ancestral (A) and Omicron variants (BA.1, and BA.5). However, with the continuous mutation and evolution of the virus, new variants such as XBB, XBB.1.5, XBB.1.16, BF.7, and BQ.1 continue to emerge. These variants may possess different stability and transmission abilities, increasing the complexity of viral mutations. The study of newer mutant strains is essential because it may aid in improving the current understanding of virus evolution and transmission. The third limitation is related to experimental conditions; all the included studies conducted experiments on the stability of SARS-CoV-2 and influenza viruses in the environment. However, they investigated the effectiveness of disinfection under controlled laboratory conditions, without entirely simulating complex factors in actual environments, such as different seasons, humidity, sunlight exposure, population density, air circulation, and cleanliness. These factors could affect the survival and transmission of viruses in the environment. Fourthly, during the paper review process, the sample collection conditions for each research group of each literature are different, and the data results are also absolutely different. Therefore, future studies must focus on overcoming these limitations to comprehensively and accurately understand the behavioral characteristics of viruses in the environment. Their results may provide an effective scientific basis for epidemic prevention and environmental cleanliness and improve the research results’ reliability and generalizability, providing more effective guidance for disease prevention and control.

Our review underscores the significance of eliminating viral contamination, which may minimize the risks of both SARS-CoV-2 and influenza virus contamination on material and skin surfaces and mitigate covert transmission of epidemics. Initially, when encountering potentially contaminated surfaces of pertinent objects (e.g., goods during logistical transportation, public vehicles, and medical facilities), proactive adoption of personal protective measures is imperative, including wearing masks and gloves and using disposable protective clothing when necessary. After a surgical procedure is completed, protective equipment should be removed promptly according to the specifications and disposed of as contaminated waste. When using places and objects potentially contaminated by viruses, taking effective disinfection measures is crucial. The use of ethanol (70%), isopropanol (70%), bleach (10%), or hydrogen peroxide (1–3%) applied through spraying or wiping should be prioritized to ensure sufficient disinfectant contact and 15–30-min exposure. Disinfectants such as PVP-I (1 mg/mL for 1 min) or cetylpyridinium chloride (0.1 mg/mL for 2 min) can be used for maintaining oral hygiene (Chen et al., 2023; Wang et al., 2021). In environments possibly contaminated by viruses (e.g., operating rooms in medical facilities or public places in disease outbreak areas), chlorine-containing disinfectants (500 mg/L) or hydrogen peroxide gas should be used for comprehensive terminal disinfection. On valuable items or special materials that cannot withstand chemical disinfectants or in public places, ultraviolet irradiation (at 222 nm) may be used. In combination with ventilation systems, ultraviolet radiation can ensure effective air purification. The comprehensive implementation of these measures may aid in minimizing viral infection spread and risk (Eslami and Jalili, 2020; Tang et al., 2020).
